# A Great River in Alaska

**Published:** 1881-04

**Authors:** 


### A GREAT RIVER IN ALASKA.

The San Francisco *News-Letter* says: When the late Mr. Seward purchased Alaska from the Czar, he was not aware of the fact that he was getting with his countless fur-seals, fisheries, mines and icebergs, one of the greatest rivers in the world, and now almost demonstated to be of greater volume than the Mississippi. Such a stream is the Yucon.

The vast region it waters remains almost as much a *terra incognita* as the Congo. In fact, while the latter has been once explored—by Stanley—from the point where Livingstone turned back down to the Atlantic Ocean, and by Livingstone from its extreme sources to where Stanley's explorations began, no traveler has ever yet seen the upper water of the Yucon, or has been able to enlighten the world as to its length or its source, or the region it drains.

Here, then, is an opening for enterprise and ambition, more fruitful of promise than anything as yet unrevealed in Africa or the Arctic Sea, and probably less dangerous. That the country contains mines of gold and silver we may readily conjecture from the fact that such mines exist on all sides of it. The river is navigable for hundreds of miles. It is free of ice from June to September. Its banks are flanked below with Indian villages. Its waters are filled with fish for the support of human life, and its woods with game. The mountains in which it rises are unknown to white men, but, as they are generally believed to be stored with that sort of treasure which led to the rapid settlement of California and to the expansion of commerce on the South and Central Pacific, there is the strongest sort of temptation on the part of thousands to see them, test them and dig them up, if the treasure can be found.

The government has many vessels lying idle and uselessly rotting for the want of action. Why not fit one of them up for a two or three years' cruise on this great unexplored river of the North? The discovery of gold mines there would lead instantly to a large migration from all parts of the world, and in a few years contribute millions to the commerce of the Southern Pacific States and Territories.

---

Pride and vanity are forever spoken of side by side, and many suppose that they are merely different shades of the same feeling.

### SHALL WE MEET AGAIN?

The following is one of the most brilliant paragraphs ever written by the lamented George D. Prentice:

The fiat of death is inexorable. No appeal for relief from that great law which dooms us to dust. We flourish and fade as the leaves of the forest, and the flowers that bloom, wither and fade in a day, having no frailer hold upon life than the mightiest monarch that ever shook the earth with his footsteps.

Generations of men will appear and disappear as the grass, and the multitude that throng the world to-day will disappear as footsteps on the shore. Men seldom think of the great event of death until the shadow falls across their own pathway, hiding from their eyes the faces of loved ones, whose living smile was the sunlight of their existence. Death is the antagonist of life, and the thought of the tomb is the skeleton of all feasts. We do not want to go through the dark valley, although its dark passage may lead to paradise; we do not want to go down into the damp graves, even with princes for bedfellows. In the beautiful dream of "Ion" the hope of immortality, so eloquently uttered by the death-devoted Greek, finds deep response in every thoughtful soul. When about to yield his life a sacrifice to fate, his Clemanthe asks if they should meet again; to which he responds, "I have asked that dreadful question of the hills that look eternal—of the clear streams that flow forever—of stars among whose fields of azure my raised spirits have walked in glory. All are dumb. But as I gaze on thy living face, I feel that there is something in love that mantles through its beauty that cannot wholly perish. We shall meet again, Clemanthe."

